# Intercellular communications in multispecies oral microbial communities

**DOI:** 10.3389/fmicb.2014.00328

**Published:** 2014-07-01

**Authors:** Lihong Guo, Xuesong He, Wenyuan Shi

**Affiliations:** School of Dentistry, University of California-Los Angeles, Los AngelesCA, USA

**Keywords:** oral microbial community, coadhesion, signaling transduction, metabolic interactions, cell-cell communication

## Abstract

The oral cavity contains more than 700 microbial species that are engaged in extensive cell–cell interactions. These interactions contribute to the formation of highly structured multispecies communities, allow them to perform physiological functions, and induce synergistic pathogenesis. Co-adhesion between oral microbial species influences their colonization of oral cavity and effectuates, to a large extent, the temporal and spatial formation of highly organized polymicrobial community architecture. Individual species also compete and collaborate with other neighboring species through metabolic interactions, which not only modify the local microenvironment such as pH and the amount of oxygen, making it more suitable for the growth of other species, but also provide a metabolic framework for the participating microorganisms by maximizing their potential to extract energy from limited substrates. Direct physical contact of bacterial species with its neighboring co-habitants within microbial community could initiate signaling cascade and achieve modulation of gene expression in accordance with different species it is in contact with. In addition to communication through cell–cell contact, quorum sensing (QS) mediated by small signaling molecules such as competence-stimulating peptides (CSPs) and autoinducer-2 (AI-2), plays essential roles in bacterial physiology and ecology. This review will summarize the evidence that oral microbes participate in intercellular communications with co-inhabitants through cell contact-dependent physical interactions, metabolic interdependencies, as well as coordinative signaling systems to establish and maintain balanced microbial communities.

## INTRODUCTION

With the respect to microbial flora, the human oral cavity is one of the most densely populated sites of the human body, consisting of as many as 600–800 bacterial species (Paster et al., 2006; Dewhirst et al., 2010). Extensive clinical studies have indicated that the oral microbial flora is responsible for two major human diseases: dental caries and periodontitis (Marsh, 1994). The environmental diversity of the oral cavity promotes the establishment of distinct microbial communities. For example, the supragingival plaque is known to be dominated by Gram-positive streptococci and the subgingival plaque is populated with mainly Gram-negative anaerobic bacteria (Marsh, 1994). These microbial inhabitants have co-evolved not only with their host, but also with each other, leading to extensive intercellular communications across species. The ecological equilibrium of a well-organized multispecies oral microbial community is maintained through competitive and cooperative interactions between microorganisms of the same or different species at the cellular and molecular levels.

Intercellular communications are essential for the development of temporal and spatial organization of oral microbial communities, and are involved in processes such as provision of attachment sites, modification of local microenvironment, cooperative nutrient utilization, as well as synergistic and competitive interactions (Kuramitsu et al., 2007). In addition, intercellular signaling initiated by cell–cell contact, quorum sensing, and other diffusible signaling molecules allow oral microbial species to coordinate their physiological behaviors and pathogenesis (Jenkinson and Lamont, 2005; Kolenbrander et al., 2006). All of these intercellular interactions between oral microbes enable them to select co-residents, promote the establishment of a highly structured and diverse microbial community, and play an essential role in microbial pathogenesis.

## PHYSICAL INTERACTIONS

The ability of species to adhere to salivary pellicles or to bacteria that are already attached to the surface is among the key factors that determine whether a species can perpetuate in the oral cavity. Initial adhesion of the early colonizers of oral microbial communities invariably involves binding to saliva components that are adsorbed to solid surfaces such as teeth or to desquamating surfaces such as epithelial tissue. Oral streptococci are believed to be among the earliest inhabitants on tooth surfaces due to their capability to adhere directly to salivary pellicle and comprise about 80% of the early colonizers (Avila et al., 2009). Meanwhile, *Actinomyces* has also been identified in the inner portion of the dental biofilms, suggesting their early colonizer nature (Palmer et al., 2003; Dige et al., 2009). Other early colonizers are comprised of *Veillonella* and *Neisseria* (Li et al., 2004). Several studies have identified adhesins and receptors for early colonizing microorganisms to adhere specifically to salivary pellicle and even determine the unique domains on these molecules involved in binding. For example, it has been shown that Hsa mediated binding of *Streptococcus gordonii* to sialic acid-containing receptors in salivary pellicle (Ding et al., 2010) and the region containing lysine in the salivary components may have binding activity to *S. gordonii* and *Streptococcus sanguinis*. Meanwhile, the scavenger receptor cysteine-rich domain peptide 2 (SRCRP 2) region may also function as a receptor for the binding of streptococci (Hamada et al., 2004).

**FIGURE 1:**
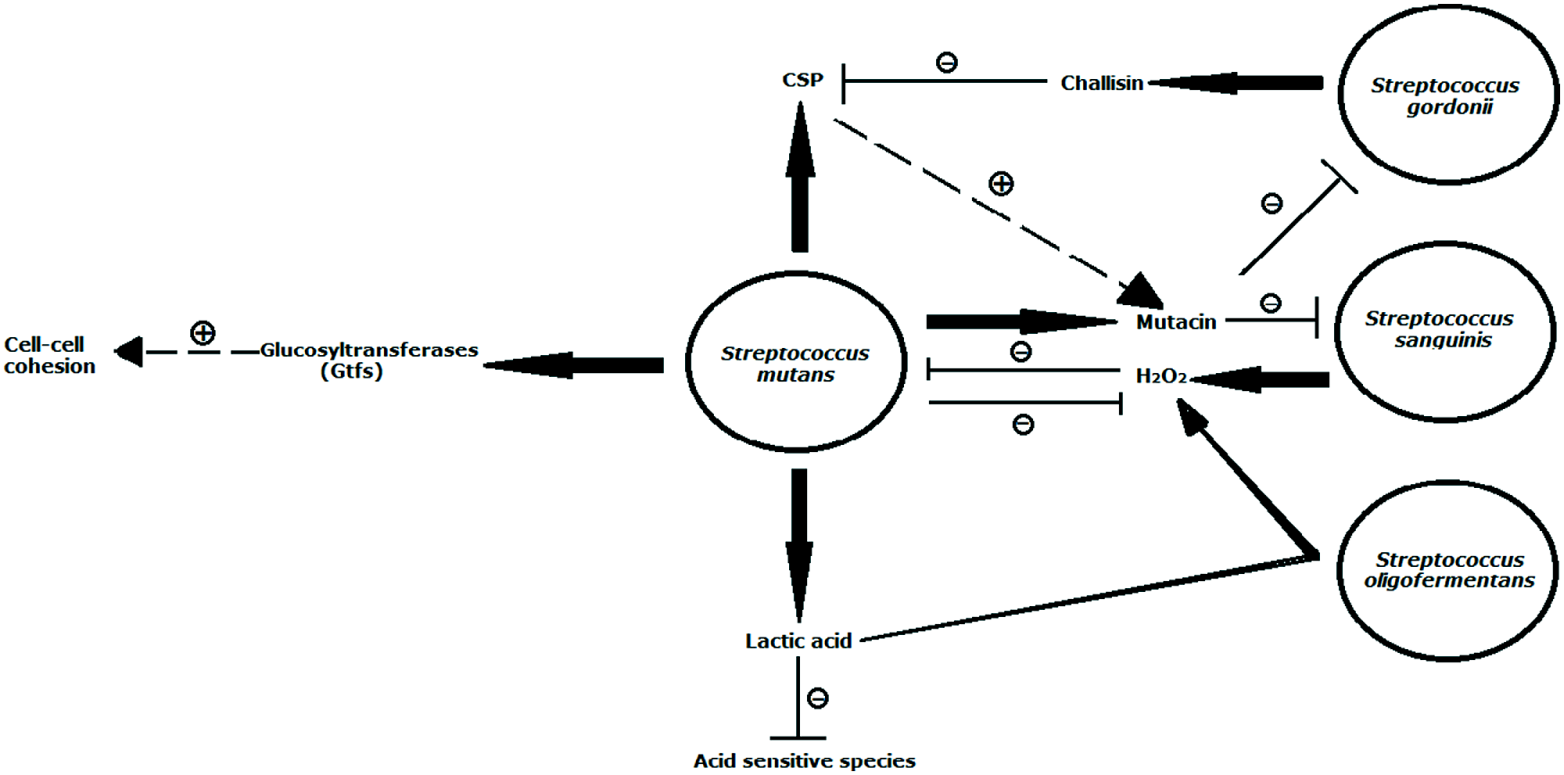
**Illustration of interspecies interactions between *Streptococcus mutans* and selected oral streptococci that have been studied on a molecular level.** Depicted is the production of lactic acid, mutacin, glucosyltransferases (Gtfs), and CSP, which also enhances mutacin production by *S. mutans*. Lactic acid has been found to inhibit proliferation of a variety of acid-sensitive anaerobe species, but it was recently discovered to serve as a substrate for *S. oligofermentans* for H_2_O_2_ production. This compound is also generated by *S. sanguinis* and was previously shown to inhibit growth of *S. mutans.* In order to counteract this antagonistic molecules, *S. mutans* produces some oxidative stress factors such as Dps-like protein (Dpr) and superoxide dismutase (Sod) in modulating its competitive fitness by alleviating H_2_O_2_-mediated interspecies competition exerted by other streptococcal co-residents. *S. sanguinis* and *S. gordoniiare* are sensitive to the mutacins produced by *S. mutans*. The Gtfs exoenzymes of *S. mutans* adhere to the cell surfaces of same species or other residents in an enzymatically active form and the glucans formed *in situ* increase cell–cell cohesion. *S. gordonii* utilizes challisin to reduce mutacin production by reducing the levels of the stimulating factor CSP. The dashed arrow in combination with + indicates stimulation, solid arrows symbolize production, and ⊣ in combination with – designates inhibition. Model is modified from Kuramitsu et al. (2007).

Biofilm matrix also plays a role in maintaining microbial colonization, therefore contributing to oral biofilm development. *Streptococcus mutans*, a predominantly prevalent caries-associated species in humans (Loesche, 1986), utilizes extracellular polysaccharides synthesized by glucosyltransferases (Gtfs) to facilitate its firm attachment to teeth and promote tight cell clustering (Koo et al., 2010; Xiao et al., 2012). The Gtfs exoenzymes of *S. mutans* were shown to adhere strongly not only to the salivary pellicle that covers the surface of teeth but also to bacterial surfaces, and could produce glucans in the adsorbed state (Vacca-Smith and Bowen, 1998; Bowen and Koo, 2011). In view of the cohesive attribute (Loesche, 1986; Tamesada et al., 2004), glucans synthesized on the pellicle provides additional bacterial binding sites, while the polymers on the cell surfaces of same species or other residents increase cell–cell cohesion (**Figure 1**). For example, GtfB, one of Gtfs, binds effectively to both yeast and hyphae cell forms of* Candida albicans* in an enzymatically active form and the glucans formed *in situ* not only enhances the binding of *S. mutans* cells to *C. albicans* cells but also promotes the colonization of *C. albicans* on the tooth surface (Gregoire et al., 2011). Among the numerous types, the rigid alpha 1, 3 linked glucans are particularly suited for cohesion (Rölla et al., 1985). Due to its important role in promoting microbial adherence and clustering, many oral bacterial species modulate their behavior within biofilm by interfering with matrix structure. *Actinobacillus* *actinomycetemcomitans*, the predominant pathogen in aggressive periodontitis was reported to be able to produce a matrix-degrading enzyme dispersin B for degrading poly-*N*-acetylglucosamine (PNAG), a major polysaccharide component of biofilm matrix, and thus, caused detachment and dispersion of *A. actinomycetemcomitans* biofilm cells (Kaplan et al., 2003). *Streptococcus salivarius* produces a fructosyltransferase (FTF) and an exo-beta-D-fructosidase (FruA) to hinder further polymicrobial community development with other oral bacteria such as *S. mutans* (Ogawa et al., 2011).

After the initial attachment of early colonizers to salivary receptors, the early colonizers could transform teeth or oral soft tissue surface to a bacterial surface. The adhesins on cell surface of early colonizers enable the sequential addition of the partner species. Oral streptococci express a diversity of cell surface molecules functioning as adhesins that recognize diverse bacterial receptors, such as pili, surface fibrils and Antigen I/II family protein, indicating that streptococci have a broad pairwise match with succeeding colonizers. It has been documented that oral streptococci recruit strains of *Actinomyces* by a highly selective cell surface interactions between specific streptococcal cell wall polysaccharides and *Actinomyces* sp. type 2 fimbriae (Cisar et al., 1979). Additionally, an inducible high-affinity Mn^2+^ transporter of *S. gordonii* (Kolenbrander et al., 1998a) was found to mediate its coadhesion with *Actinomyces naeslundii* (Kolenbrander et al., 1994). The close spatial association between streptococci and *Actinomyces*, as well as between streptococci and *Veillonella* was visualized in early stage of dental biofilms *in vivo* (Palmer et al., 2003; Chalmers et al., 2007).

The bacteria that can recruit subsequent colonizing species through binding to early and late colonizers constitute middle colonizers. *Fusobacterium nucleatum* is considered a “bridge-organism” that links early commensal colonizers and late colonizing species including periodontal pathogens and is important in the succession of genera in oral multispecies communities. Multivalent adhesins on the cell surface of *F. nucleatum*, with their distinct selectivity to early and late colonizers, contribute greatly to the development of polymicrobial communities within the oral cavity. It has been shown that the distinctive corncob structure visible in mature plaque is formed by the coadhesion between *F. nucleatum* and streptococci (Lancy et al., 1983). In addition to streptococci, other early colonizers such as *A. naeslundii* and *Veillonella parvula* also bind to *F. nucleatum* (Biyikoǧlu et al., 2012). Among the multiple adhesins of *F. nucleatum*, an arginine-inhibitable adhesin (RadD) is responsible for its adherence to streptococci; while some serotype-specific adhesins are involved in binding to periodontopathogens including *Porphyromonas gingivalis*, *A. actinomycetemcomitans* and *Treponema denticola* (Rosen and Sela, 2006; Rosen et al., 2008; Rupani et al., 2008; Kaplan et al., 2009). The coadhesion between *F. nucleatum* and these late colonizing Gram-negative species are usually mediated by lectin–carbohydrate interactions. For instance, the recognition between a galactose-specific lectin-like adhesin expressed by *F. nucleatum* and the sugar moiety present in the capsule and lipopolysaccharide of* P. gingivalis* contributes to coadhesion between the two species (Rosen and Sela, 2006). Similar galactose-containing receptors are also found on cell surface of *A. actinomycetemcomitans* (Rupani et al., 2008) and *T. denticola* (Rosen et al., 2008). As for *Tannerella forsythia* which is associated with severe and chronic periodontitis, its cell surface glycoproteins in S-layer act as adhesins in coadhesion with *F. nucleatum* (Shimotahira et al., 2013). Apart from protein–carbohydrate interactions, some researchers have reported that leucine-rich repeat in proteins from *T. denticola* and *T. forsythia* are involved in protein–protein interactions with each other and with *F. nucleatum* (Ikegami et al., 2004; Sharma et al., 2005). Even though *T. forsythia* possesses an S-layer at the outermost cell surface, a portion of the protein is exposed on the cell surface, one of them being OmpA-like protein, which was proposed to play a role in intercellular adhesion (Abe et al., 2011). Through selective recognition of adhesins on cell surface of *F. nucleatum*, these periodontitis associated species are integrated into microbial communities. For example, *Filifactor alocis*, an emerging important periodontal pathogen was found to preferentially accumulate at sites rich in *F. nucleatum* (Wang et al., 2013).

Besides *F. nucleatum*, *P. gingivalis* also exhibits a broad range of pairwise interactions with other microbial community members. *P. gingivalis* was shown to be able to assemble into a heterotypic microbial complex with *S. gordonii* and *F. nucleatum* (Lamont et al., 2002; Periasamy and Kolenbrander, 2009b). The specific molecular interactions between *P. gingivalis* and *S. gordonii* have been well characterized and occur through Mfa-Ssp and fimbrial adhesin (FimA)-GAPDH adhesin–receptor pairs (Maeda et al., 2004; Kuboniwa and Lamont, 2010). *P. gingivalis*’ short fimbriae (Mfa) bind to *S. gordonii* AgI/II family proteins SspA and SspB (Lamont et al., 2002; Park et al., 2005), whereas *P. gingivalis* long fimbriae (FimA) mediate its attachment to glyceraldehyde-3-phosphate dehydrogenase (GAPDH) of *Streptococcus cristatus* (Maeda et al., 2004). Further researches revealed that the region defined by residues 1167–1250 of *S. gordonii* SspB protein was essential motif for *P. gingivalis* binding (Brooks et al., 1997), and both NITVK motif and the nuclear receptor box of SspB contributed to the Mfa–SspB interaction (Demuth et al., 2001; Daep et al., 2011). The binding domain of GAPDH present on the streptococcal surface specific for *P. gingivalis* long fimbriae exists within the region encompassing amino acid residues 166–183 of GAPDH (Nagata et al., 2009). Although Mfa and FimA participate in the coadhesion of *P. gingivalis* to streptococci, another *P. gingivalis* surface molecule InlJ retards the development of *P. gingivalis–S. gordonii* community (Capestany et al., 2006), which allows *P. gingivalis* to fine-tune the extent of buildup of microbial communities. Besides, Kuboniwa et al. (2009) reported that *P. gingivalis* had a decreased ability to partner with *S. gordonii* and *F. nucleatum* when being defective in HmuR, a major hemin uptake protein. Consistent with the interspecies interaction described above, biofilm visualization by confocal microscopy confirmed that *P. gingivalis* colonized *in vitro* biofilms mainly within regions where *S. gordonii* accumulated (Kuboniwa et al., 2006).

Late colonizers are species with weak colonizing ability that require partner species to integrate into microbial communities and are comprised primarily of anaerobic, Gram-negative bacteria (Kolenbrander et al., 2006; Zijnge et al., 2010). As a late colonizer, *T. denticola* is unable to adhere to oral surfaces such as teeth or epithelial tissue; however, in the presence of *P. gingivalis*, it could colonize oral biofilms. In sub-gingival dental biofilms, *T. denticola* is always found more external relative to *P. gingivalis* (Ishihara et al., 2004). The chymotrypsin-like proteinase (CTLP), found within a high-molecular-mass complex on the cell surface of *T. denticola*, mediates its adherence to other potential periodontal pathogens including *P. gingivalis*, *F. nucleatum*, *Prevotella intermedia* and *Parvimonas micra*, and thus is crucial for *T. denticola* to integrate into multispecies oral microbial communities (Cogoni et al., 2012).

## PHYSIOLOGICAL INTERACTIONS

### METABOLITES MEDIATED COOPERATION

The high cell density of the oral microbial community impels individual resident to compete and collaborate with its neighboring species in order to survive in a hostile environment. An important factor in determining the microbial colonization in multispecies communities is the availability of nutrients. Metabolic communications among oral microbes may occur through the excretion of a metabolite by one bacterium that can be utilized as a nutrient by another species (Takahashi et al., 2010; Liu et al., 2012), or through syntrophic biochemical enzymes to cooperatively metabolize a substrate (Kolenbrander et al., 2002).

**FIGURE 2:**
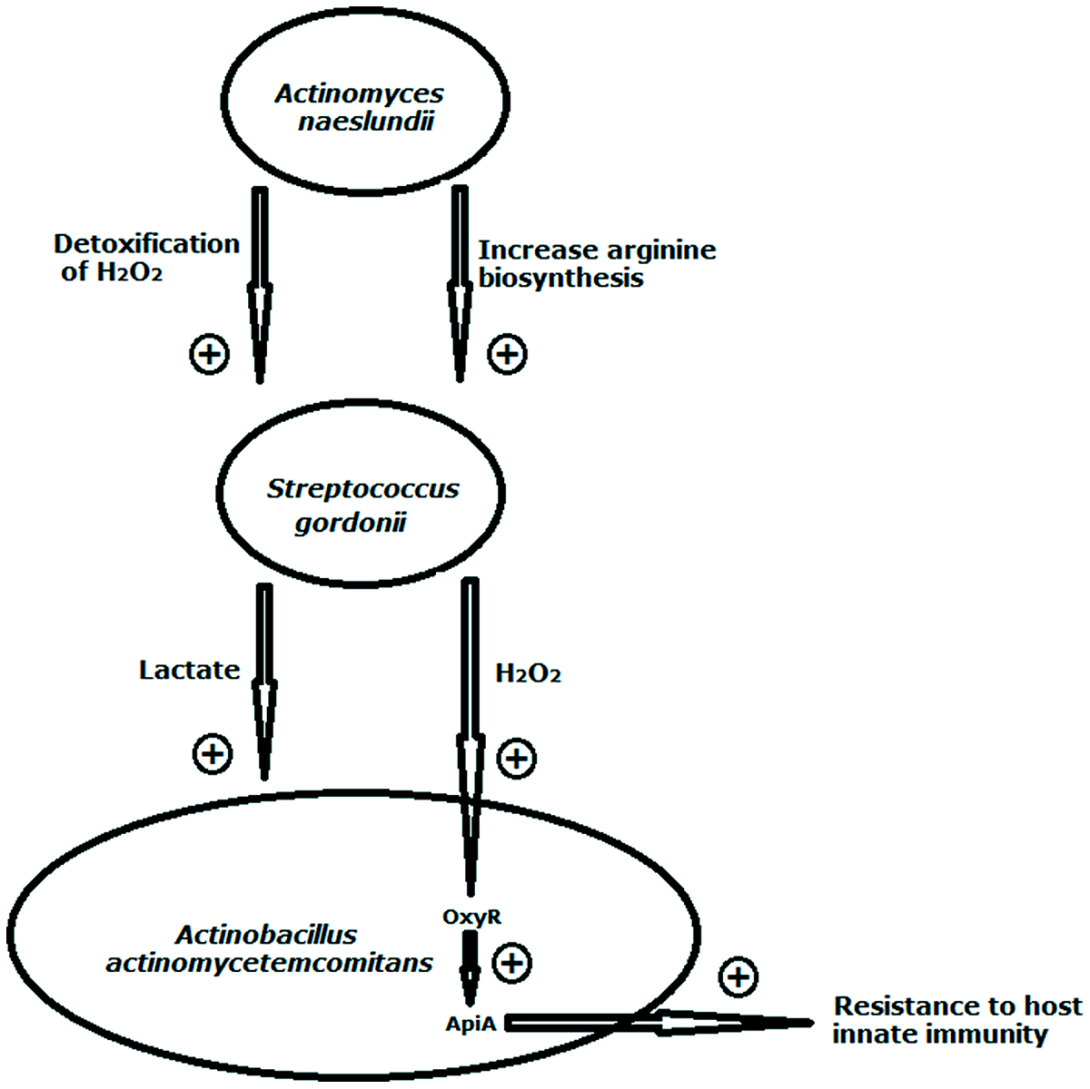
**Illustration of interspecies interactions between *Streptococcus gordonii* and its co-residents *Actinomyces naeslundii* and *Actinobacillusactinomycetemcomitans*.** *S. gordonii* benefits from its coaggregation with *A. naeslundii* both in tolerating protein oxidation by H_2_O_2_ and in upregulating arginine biosynthesis. *A. actinomycetemcomitans* utilizes L-lactate, a carbohydrate metabolic by-product of *S. gordonii* as nutrient source. The consumption of lactic acid by the lactic acid-utilizing bacteria minimizes lactic acid accumulation, which otherwise would inhibit streptococcal growth. *S. gordonii*-generated H_2_O_2_ can stimulate activation of the *A. actinomycetemcomitans* OxyR regulator to increase transcription of *apiA* that leads to enhanced resistance to host innate immunity. The arrow in combination with + indicates stimulation. Model compiled from Brown and Whiteley (2007), Jakubovics et al. (2008a, b), and Ramsey and Whiteley (2009).

In the oral cavity, the short-chain acids such as lactate and acetate produced by early colonizers *via* sugar metabolism serve as carbon and energy sources for succeeding colonizers. This cross-feeding ensures sequential microbial colonization (Kolenbrander, 2011). Oral streptococci are well known by its ability to generate lactic acid as a by-product of sugar fermentation, whereas some neighboring species, such as *Veillonellae* sp. (Kumar et al., 2005; Chalmers et al., 2008) are unable to ferment sugars, but use lactic acid as a preferred source of carbon and energy. The ability of *A. actinomycetemcomitans* to utilize L-lactate, a carbohydrate metabolic by-product of *S. gordonii* as nutrient source allows it to integrate and become much more competitive within oral polymicrobial communities (Brown and Whiteley, 2007; Ramsey et al., 2011; **Figure 2**). The consumption of lactic acid by these lactic acid-utilizing bacteria minimizes lactic acid accumulation, which otherwise would cause streptococci to repress amylase synthesis and reduce the utilization efficiency of fermentable sugars by both partners (Egland et al., 2004). *P. gingivalis* and *T. denticola* are also metabolically connected. Succinate produced by *T. denticola* supports the growth of *P. gingivalis*, which, in turn, enhances the growth of *T. denticola* by providing isobutyric acid and proteinaceous substrates (Grenier, 1992; Nilius et al., 1993). Besides short-chain acids, *Porphyromonas* sp. can acquire vitamin K-like growth factors from *Veillonella* (Hojo et al., 2009).

Another type of metabolic interaction involves the complementary substrate utilization. Individual species in the oral cavity usually do not contain complete metabolic pathways to degrade complex salivary components. However, the breakdown of these compounds can be achieved through cooperation among multiple species. The synergistic and concerted action of *Streptococcus mitis*, *S. gordonii*, *Streptococcus crostatus*, and *A. naeslundii* with overlapping patterns of enzyme activity in salivary mucin catabolism has been reported (Wickström et al., 2009). Similarly, co-culture of *S. sanguinis* and *S. oralis* obtains significantly higher degradation efficiency of mucin than in monospecies culture (van der Hoeven and Camp, 1991). Therefore, this type of metabolic cooperation can benefit the participating species through maximizing their potential to extract energy from limited substrates.

Metabolic synergistic partnership usually occurs among community residents that often co-localize within the same niche and are metabolically compatible. In a three-species flow cell, *P. gingivalis* could not grow together with *Streptococcus oralis–A. actinomycetemcomitans* and *S. oralis*–*F. nucleatum* two-species community but displayed mutualistic growth when paired with *S. gordonii*–*S. oralis*, or *V. parvula*–*A. actinomycetemcomitans*, as well as with *V*. *parvula*–*F. nucleatum* two-species community (Periasamy and Kolenbrander, 2009a, b). The results indicated that *P. gingivalis* and *S. oralis* are incompatible, whereas *S. gordonii* could assist *P. gingivalis* to overcome this incompatibility, which also indicated that *S. oralis* was much more restricted in its successful partnership compared to its relative *S. gordonii*. Furthermore, in the presence of *V. parvula*, *S. oralis* displays positive partnership either with *P. gingivalis* or *F. nucleatum*. These findings illustrate the high species selectivity during multispecies community development.

### METABOLITES MEDIATED COMPETITION

Interspecies antagonism reflects the competition among bacterial species for limited space and nutritional resources, and is the fundamental driving force in defining the structure and activity of polymicrobial oral communities. H_2_O_2_ secreted by viridans streptococci, including *S. sanguinis*, *S. oralis*, *S. mitis*, *S. gordonii*, *Streptococcus parasanguinis*, and a few *S. mutans* strains, plays an important role in interspecies competitive interaction (**Figure 1**). An inverse correlation between *Streptococcus oligofermentans* and *S. mutans* has been reported and thus suggested a possible antagonistic interaction between the two species (Tong et al., 2003). Tong et al. (2007) showed that *S. oligofermentans* possesses the capacity to convert its competitor’s main “weapon” (lactic acid) into an inhibitory chemical (H_2_O_2_) against *S. mutans* in order to gain a competitive growth advantage. This fascinating ability may be an example of a counteroffensive strategy used during chemical warfare within the oral microbial community. Furthermore, the inhibitory nature of H_2_O_2_ helps to structure the microbial composition of multispecies communities and contributes to an overall ecological homeostasis. It has been known that high levels of cariogenic *S. mutans* in the oral cavity correlate with low levels of *S. sanguinis* (Caufield et al., 2000). An epidemiological survey demonstrated the presence of putative periodontopathogens such as *P. gingivalis*, *A. actinomycetemcomitans*, and *Eikenella corrodens* in a destructive periodontitis site was correlated with the absence of certain viridans streptococci including *S. sanguinis* and* Streptococcus intermedius*, and vice versa (Hillman et al., 1985).

In addition to H_2_O_2_, bacteriocin or bacteriocin-like activities in multispecies communities play an essential role in modulating the ecological balance of oral microbial communities (**Figure 1**). Some oral commensal streptococci, such as *S. sanguinis*, *S. gordonii*, and *S. oligofermentans*, can suppress the overgrowth of cariogenic species *S. mutans* through the production of bacteriocins (Kreth et al., 2005a; Heng et al., 2007; Tong et al., 2008). *S. mutans*, in turn, gains competitive edge by producing lactic acid and mutacin to antagonize the growth of other oral commensal streptococci (Loesche, 1986; Qi et al., 2001). *Lactobacillus paracasei* can also produce bacteriocin to inhibit the growth of *P. gingivalis*, *P. intermedia*, *T. forsythia*, *S. salivarius*, and *S. sanguinis* (Pangsomboon et al., 2006, 2009). *Prevotella nigrescens* (*P. nigrescens*) ATCC 25261 was reported to produce a novel bacteriocin Nigrescin against *P. gingivalis*, *P. intermedia*, *T. forsythia*, and *Actinomyces* sp. (Kaewsrichan et al., 2004). More recently, it has been suggested that *P. gingivalis* gingipains secreted in the subgingival environment are the main inhibitor in promoting the detachment of antecedent *A. actinomycetemcomitans* cells from the surface (Takasaki et al., 2013).

Some species have evolved specific mechanisms to counteract the antagonistic molecules such as H_2_O_2_ and bacteriocin (**Figure 1**). For example, *S. mutans* produces Dps-like protein (Dpr), superoxide dismutase (Sod) and glutathione synthetase (GshAB) in modulating its competitive fitness by alleviating H_2_O_2_-mediated interspecies competition exerted by other streptococcal co-residents, thus promoting its survival and persistence within the oral microbial communities (Fujishima et al., 2013; Zheng et al., 2013). Meanwhile, *S. mutans* expresses a cell-envelop associated eukaryotic serine/threonine protein kinase (STPK) to resist peroxide (Zhu and Kreth, 2010). Another example is that *S. gordonii* secrets challisin to inhibit bacteriocin production through interfering with competence-stimulating peptide (CSP)-dependent quorum sensing system in *S. mutans* (Wang and Kuramitsu, 2005).

### pH/OXYGEN MEDIATED INTERACTIONS

The co-inhabitants within the oral microbial community usually favor mutual growth in harsh conditions by modifying surrounding pH or oxygen tension. For example, as pH drops caused by acid-producing bacteria such as streptococci and actinomyces following sugar fermentation, acid-tolerant bacteria, including *S. mutans* and lactobacilli, will out-compete acid-sensitive bacteria and have great impact on the microbial composition within community (**Figure 1**). Meanwhile, the lactic acid-utilizing bacteria such as *Veillonella atypica* and *A. actinomycetemcomitans* could prevent the acid buildup in dental biofilms, thus restoring microenvironment pH toward neutrality even in the presence of lactic acid bacteria and fermentable carbohydrates. As a result, the acid-sensitive species such as *P. gingivalis* are protected from acid attack (Takahashi, 2003). Acid-neutralizing activity could also be derived from the decrease in acidity during the fermentation of amino acid into organic acids and ammonia by certain periodontopathogens (Takahashi, 2003). For instance, it has been shown that *F. nucleatum* can grow at a wide pH range of 5.0–7.0, while *P. gingivalis* is susceptible to pH below 6.5. A study by Takahashi (2003) demonstrated that *F. nucleatum* can generate ammonia from glutamic and aspartic acid, subsequently elevating the pH to favor *P. gingivalis* growth. Besides adjusting local pH, *F. nucleatum* is also aerotolerant and its metabolic activity can reduce the concentration of oxygen to levels that can be tolerated by obligate anaerobes such as *P. gingivalis*, *P. Nigrescens*, and *T. denticola* (Bradshaw and Marsh, 1998; Bradshaw et al., 1998; Kolenbrander et al., 1998b). Without *F. nucleatum*, the number of obligate anaerobes would decrease sharply in oxygenated environments (Bradshaw et al., 1998). Meanwhile, *F. nucleatum* can support *P. gingivalis* growth by providing a capnophilic microenvironment when growing in a CO_2_ depleted environment (Diaz et al., 2002).

### DNA MEDIATED INTERACTIONS

Cell lysis resulting from H_2_O_2_ and bacteriocin has been conjectured to provide genomic DNAs to the microbial communities (Zhu and Kreth, 2012). Extracellular DNA can be trapped within microbial biofilm matrix (Nadell et al., 2009) and might help to stabilize the biofilm structure (Barnes et al., 2012). Perry et al. (2009) found that treating *S. mutans* biofilms with DNase I reduced the surface-associated biomass by approximately 20%.

Genetic exchange occurs within oral microbial communities because residents are in close proximity. Horizontal gene transfer is the main driving force in bacterial evolution of antibiotic resistance and pathogenicity (Roberts and Mullany, 2010). Genome sequencing of several oral bacteria has revealed the presence of “pathogenicity islands” on the chromosomes of species such as *P. gingivalis* (Curtis et al., 1999). Conjugation, transformation, and transduction have been commonly observed in a variety of oral bacterial species (LeBlanc et al., 1978; Kuramitsu and Trapa, 1984; Yeung, 1999; Li et al., 2001b). Many genera of oral bacteria, including *Actinomyces*, *Bifidobacterium*, *Fusobacterium*, *Haemophilus*, *Peptostreptococcus*, *Streptococcus*, and *Veillonella* contain conjugative transposons that facilitate intercellular DNA transfer through conjugation (Rice, 1998). It is well known that oral streptococci are naturally competent, and it is possible that the DNA in the extracellular matrix is transmitted among them (Warburton et al., 2007). A study reported by Kreth et al. (2005b) proposed that *S. mutans* might utilize competence-induced bacteriocin to kill and lyse the neighboring species colonizing the same ecological niche and take up their DNA. Besides streptococci, *P. gingivalis* is able to exchange fimbrial allele types I and IV via natural competence as an adaptive process to modify behavior (Kerr et al., 2014). *A. actinomycetemcomitans* isolates from periodontal pockets have also been found to contain bacteriophages (Sandmeier et al., 1995).

## MICROBIAL INTERCELLULAR SIGNALING

Within dense and constricted oral microbial community, intercellular signaling allows residents to regulate their gene expressions in accordance with their environments, and coordinate their behaviors in virulence factors regulation, metabolic adaptation, competence initiation and polymicrobial biofilm development.

### CONTACT-DEPENDENT SIGNALING

In response to physical association with host tissues or bacterial cell surface, bacteria could initiate signal transduction cascades to regulate the expression of genes involved in adherence (Zainal-Abidin et al., 2012; Sarkar et al., 2014). For example, attachment of *S. gordonii* to salivary pellicle on the tooth surface triggers upregulated expression of AgI/II family protein Ssp (Dû and Kolenbrander, 2000), which are adhesins to salivary proteins (Demuth et al., 1996), as well as *Actinomyces* and *P. gingivlais* (Lamont et al., 2002). Further study revealed that this transcriptional regulation was mediated by a two-component system BrfAB (Zhang et al., 2004). Thus, the direct contact with saliva enhances *S. gordonii’s* adherence to tooth surface and the recruitment of late colonizing species such as *Actinomyces*, which will contribute to the spatiotemporal development of multispecies communities. Meanwhile, signaling can also be triggered when different bacterial species are in direct physical contact. *P. gingivalis* was reported to acquire increased adhesive capacities on various substrata through up-regulated expression of gingipain upon contact with *T. denticola* (Meuric et al., 2013). The transcriptional responses as a result of cell–cell contact often are not limited to a few genes. A study showed that thirty-three genes were differentially regulated during accretion of *P. gingivalis* in heterotypic biofilms with *S. gordonii* (Simionato et al., 2006). The functions of the regulated genes were predominantly related to metabolism and energy production. Another study on *P. gingivalis* assembled into a mixed community with *F. nucleatum* and *S. gordonii* revealed that over 400 genes were differentially regulated in response to cell–cell contact (Kuboniwa et al., 2009). Such signaling is important in the integration of *P. gingivalis* into early biofilms dominated by Gram-positive bacteria. In addition to facilitating bacteria colonization of microbial community, contact-inducible gene expression could also enhance microbial growth. *S. gordonii* was shown to benefit from its coaggregation with *A. naeslundii* in arginine-restricted conditions through three fold changes in the expression of the genes involved in arginine biosynthesis (Jakubovics et al., 2008a; **Figure 2**). This signaling was not observed in co-cultures where there was no coaggregation between the two species, suggesting that there is a specific transcriptional response after cell–cell contact. Contact-inducible transcriptional change could prepare bacteria to better cope with the environmental stress, such as the exposure to H_2_O_2_ within the multispecies communities. An example comes from our observation of *S. sanguinis* and *F. nucleatum* (He et al., 2012). We found that upon contact with *S. sanguinis*, *F. nucleatum* acquired increased resistance to H_2_O_2_ and also significantly inhibited H_2_O_2_ production by *S. sanguinis*. This event was not observed in a *F. nucleatum* mutant deficient in *radD*, which encodes an outer membrane protein adhesin responsible for coadhesion with streptococci. This defense strategy of *F. nucleatum* prevents antagonism by other oral bacteria and allows integration into the developing oral microbial community. Similarly, coaggregation with *A. naeslundii* protects *S. gordonii* from oxidative damage by H_2_O_2_ (Jakubovics et al., 2008b; **Figure 2**).

On the other hand, cell contact-dependent signaling has also been shown to reduce adhesion. Park et al. (2006) observed that following contact with *S. gordonii*, the short fimbriae adhesin Mfa of *P. gingivalis* was down-regulated. Moreover, a cascade of tyrosine phosphorylation/dephosphorylation events in *P. gingivalis* initiated by contact with *S. gordonii* constrains the heterotypic community development between the two species (Maeda et al., 2008; Chawla et al., 2010). Similarly, cell–cell contact between *T. forsythia* and *F. nucleatum* or *T. forsythia* and* P. gingivalis* also initiates a signal transduction cascade that causes down-regulated expression of BspA leucine-rich repeat protein adhesin in *T. forsythia* (Inagaki et al., 2005), resulting in reduced adherence ability. There is a signaling down-regulation in microbial biosynthesis pathway as well. A study showed *S. gordonii* and *F. nucleatum* reduced the abundance of *P. gingivalis* proteins responsible for several vitamin synthesis and pyrimidine biosynthesis in a contact-dependent manner (Kuboniwa et al., 2009).

Some degree of specificity exists in response to contact-dependent signaling. The study of Hendrickson et al. (2012) demonstrated that, when in contact with *P. gingivalis*, *S. gordonii* up-regulates the expression of the glycolysis pathway while when in contact with *F. nucleatum* the pentose phosphate pathway is up-regulated.

### QUORUM SENSING

In addition to contact-dependent signaling, microbial intercellular signaling can also be mediated by secreted diffusible molecules. Quorum sensing mediated by autoinducing diffusible molecules (autoinducer) is a system of stimulus and response correlated to population density, which allows bacteria to coordinate gene expression on a population-wide scale. A significant portion of a bacterial genome (4–10%) and proteome (20% or more) can be influenced by quorum sensing signaling (Dunman et al., 2001; Arevalo-Ferro et al., 2003; Schuster et al., 2003; Wagner et al., 2003). Common classes of autoinducer are *N*-Acyl Homoserine Lactones (*N*-AHL) found in Gram-negative bacteria, autoinducing peptides (AIPs) produced by Gram-positive bacteria, and a family of autoinducers known as autoinducer-2 (AI-2) which is widespread among both Gram-negative and Gram-positive bacteria (Miller and Bassler, 2001) and is known as the most conserved quorum sensing signal molecule.

In Gram-negative bacteria, *N*-AHL signal is mainly generated by an *N*-AHL synthase of the LuxI family of proteins, and is recognized by an *N*-AHL receptor protein belonging to the LuxR family of transcriptional regulators (Ryan and Dow, 2008). Most Gram-negative bacteria use analogous LuxI/LuxR-type circuits for intraspecies signaling in which only members of the same species are able to recognize and respond to the signal. Meanwhile, there are reports suggesting their involvement in interspecies signaling (Huber et al., 2001; Riedel et al., 2001). The* N*-AHL intra- and interspecies signaling role of oral microbes needs to be further investigated. In this review we mainly focus on cell–cell signaling *via* AIPs and AI-2.

#### AIPs

In Gram-positive bacteria, recognition of and response to the AIPs occur not by direct binding to a cognate receptor but through a two-component signal transduction system, in which AIPs binds to a membrane-bound histidine kinase sensor and the binding leads to phosphorylation of response regulator proteins that ultimately bind to the promoter of target genes to regulate gene expression (Senadheera and Cvitkovitch, 2008).

Competence-stimulating peptide-mediated quorum sensing has been identified in various oral streptococcal species including *S. mutans, S. gordonii*, and* S. intermedius* (Håvarstein et al., 1997; Cvitkovitch, 2001; Petersen et al., 2004). The proposed role of CSP-mediated quorum sensing is to alter genes transcription and proteins synthesis involved in biofilm formation, competence development, bacteriocin synthesis, stress resistance, and autolysis (Petersen et al., 2004; Senadheera and Cvitkovitch, 2008; Perry et al., 2009; Senadheera et al., 2009).

Although AIPs are generally species-specific and sometimes strain-specific, they can be detected by and induce response in other species. A study showed that *S. mutans* CSP could be sensed by *C. albicans* and resulted in induction of its germ tube formation (Jarosz et al., 2009). In *Enterococcus faecalis* (*E. faecalis*), Fsr quorum-sensing system exerts inhibitive effect on *C. albicans* hyphal morphogenesis (Cruz et al., 2013). These two observations demonstrate the potential signaling role of AIPs in interkingdom communication. In order to perpetuate in complex oral multispecies community, microbial species can contend with its competitor by decreasing the concentration of the extracellular AIPs secreted by the competitor. *P. gingivalis* was found to be able to inactivate *S. mutans* CSP and abolish CSP-induced natural transformation (Wang et al., 2011a). Wang and Kuramitsu (2005) reported that *S. gordonii*, *S. sanguinis*, *S. mitis*, and *S. oralis* inhibited *S. mutans*’ mutacin production by degrading its CSP. *S. gordonii* can secrete serine protease challisin to inactivate *S. mutans* CSP, subsequently impairing *S. mutans* biofilm formation (Wang et al., 2011b; **Figure 1**). *S. salivarius* was also reported to strongly inhibit *S. mutans* biofilm formation *via* inactivating CSP produced by *S. mutans*, which provides a competitive advantage to *S. salivarius* against its competing bacteria (Tamura et al., 2009).

#### AI-2

AI-2 is a group of molecules derived from spontaneous rearrangement of *S*-4,5-dihydroxy-2,3-pentanedione (DPD), whose production is the catabolism of *S*-adenosylhomocysteine by conserved LuxS (Schauder et al., 2001b). It is widely distributed in Gram-positive and Gram-negative bacteria and plays a widespread role in bacterial communication across species boundaries (Hardie and Heurlier, 2008; Jayaraman and Wood, 2008). AI-2 has been well-characterized as a universal interspecies signal molecule in regulating formation of multispecies biofilms (Bassler et al., 1997; Schauder and Bassler, 2001a; Whitmore and Lamont, 2011). For example, mixed biofilm formation by *A. naeslundii* and *S. oralis* in flowing saliva was shown to be dependent on the production of AI-2 by *S. oralis* (Rickard et al., 2006). A *luxS* mutant of *S. oralis*, which cannot produce AI-2, does not form dual-species biofilms with *A. naeslundii*. This defect can be restored with either genetic complementation or supplementation with synthetic AI-2. *Streptococcus*-derived AI-2 can also regulate gene expression in *P. gingivalis* (Chung et al., 2001). Meanwhile, *S. gordonii* and *F. nucleatum* can sense *P. gingivalis* AI-2 as well and translate the signal into a biofilm phenotype (McNab et al., 2003; Saito et al., 2008). In addition to streptococcal AI-2, *P. gingivlais* can response to AI-2 signal secreted by *A. actinomycetemcomitans* (Fong et al., 2001). AI-2 secreted by *F. nucleatum* is able to elicit changes in the expression of the representative adhesins of the so-called “red-complex” (*P. gingivalis*, *T. denticola*, and *T. forsythia*), and thus, increase the colonization of these periodontopathogens in oral multispecies biofilms (Jang et al., 2013a).

The concentration of AI-2 is critical for interspecies mutualism. It has been reported that above and below optimal AI-2 concentration dual-species biofilm formation of *S. oralis* and *A. naeslundii* was suppressed (Rickard et al., 2006). *F. nucleatum*, a middle colonizer of oral microbial communities, applies differential regulation of AI-2 on biofilm growth of two oral streptococci by exerting a stimulatory effect on *S. gordonii* and an inhibitory effect on *S. oralis* (Jang et al., 2013b). Cuadra-Saenz et al. (2012) found that *S. gordonii* produce more AI-2 than *S. oralis*. They suggested that AI-2 could alter the structure and composition of pioneering oral streptococcal population, thereafter influencing the subsequent succession of other species into oral microbial communities (Cuadra-Saenz et al., 2012). Commensal bacteria such as *S. oralis* and *A. naeslundii* have been shown to respond to AI-2 levels that are below those produced by species associated with oral disease, for example *F. nucleatum* (Frias et al., 2001). Commensal species produce and respond to AI-2 signals at picomolar concentrations, which are much lower than that of periodontopathogens (Kolenbrander et al., 2006). *Fusobacteria* are usually detectable in oral microbial biofilms after 8 h of biofilm development (Li et al., 2004). As entry of *Fusobacteria* increases on the tooth surface and local AI-2 concentration rises, *Fusobacteria* would initiate signal-dependent changes in gene expression regulated by AI-2. By contrast, commensal bacteria are inhibited by the increasing concentration of AI-2. Accordingly, high levels of AI-2 increase the accumulation of periodontopathogens and reduce the growth of commensal bacteria. Considering the different AI-2 concentrations, some researchers propose AI-2 as a modulator for multispecies microbial communities in transition from a beneficial community to a pathogenic community (Kolenbrander et al., 2010).

In addition to interspecies interaction, AI-2 has been reported to play a role in interkingdom signaling. *C. albicans* can sense AI-2 produced by *S. gordonii* to induce numerous transcriptional changes, including increased production of hyphae and the activation or repression of three mitogen-activated protein kinases involved in morphogenetic switching (Bamford et al., 2009).

During the development of highly organized microbial communities, individual species must be able to overcome antagonistic effects from other community co-inhabitants in order to perpetuate in the multispecies community (Vollaard and Clasener, 1994; He et al., 2012). Xavier et al. (2007) found that some species of enteric bacteria can degrade AI-2 signaling and interfere with other competing species’ ability to assess and respond correctly to changes in cell population density. No such interactions have yet been observed for oral microbial community members.

Recent studies also have demonstrated an emerging role for AI-2 as an intraspecies signal molecule besides its well-characterized function in interspecies signaling. In *A. actinomycetemcomitans* and *S. mutans*, AI-2 is required in modulating their biofilm development and virulence (Merritt et al., 2003; Shao et al., 2007; Torres-Escobar et al., 2013). While in *P. gingivalis*, AI-2 regulates iron (hemin) acquisition (James et al., 2006) and oxidative stress-related genes (Yuan et al., 2005).

### NON-AUTOINDUCING MOLECULES DEPENDENT SIGNALING

In addition to autoinducers, oral microbial species can sense and respond to extracellular non-autoinducing diffusible molecules to achieve gene regulation. It has been shown that a diffusible molecule dependent signaling exists between *S. gordonii* and *V. atypica* besides their cross-feeding through lactic acid. *S. gordonii* responds to maltose or a related sugar from the lipopolysaccharide released by *V. atypica* by up-regulating the expression of streptococcal transcription factor CcpA, which is required for amylase synthesis (Egland et al., 2004; Johnson et al., 2009). Increased amylase activity on a starch substrate causes *S. gordonii* to generate more lactate, the primary energy source for *V. atypica*. Thus, one or both species involved in this event benefit from this metabolic requirements-driven interspecies interaction. Furthermore, this type of signaling occurred only in *S. gordonii* cells located within a few micrometers of *V. atypica*, emphasizing that diffusible signals between species are designed to function over very short distances, on the order of 1 μm. In addition to coordinative effect, metabolites can induce antagonistic signaling as well. For example, extracellular arginine deiminase of *S. cristatus* and* S. intermedius* downregulates expression of FimA of *P. gingivalis*, and consequently *P. gingivalis* cannot integrate into communities together with these organisms (Xie et al., 2007; Christopher et al., 2010). In contrast, lack of the similar signaling pathway in *S. gordonii* makes it more compatible with *P. gingivalis* compared to *S. cristatus* and* S. intermedius*.

Acidic pH and H_2_O_2_ could play a role in interspecies signaling, which may affect gene expression patterns of bacteria cells growing within oral microbial communities (Li and Burne, 2001a; Deng et al., 2007). Under low microenvironmental pH, 14% of the *S. mutans* genome displays altered gene expression including quorum sensing systems (Gong et al., 2009). In response to H_2_O_2_-induced oxidative stress, there were three major protein systems of *F. nucleatum* changed (Steeves et al., 2011). *S. gordonii*-generated H_2_O_2_ can stimulate activation of the *A. actinomycetemcomitans* OxyR regulator to increase transcription of *apiA* that leads to enhanced resistance to host innate immunity (Ramsey and Whiteley, 2009; **Figure 2**).

Other than growth inhibition, antibiotics at sub-inhibitory concentration were reported to be potent global regulators of transcription for bacteria within microbial communities (Yim et al., 2006). In *S. mutans* GS5, low concentrations of antibiotics could up-regulate expression of bacteriocin immunity protein gene, which affected its sensitivity to a variety of antimicrobial agents (Matsumoto-Nakano and Kuramitsu, 2006).

## CONCLUSION

Recently, there is an increasing appreciation of the essential roles of inter-cellular communications in polymicrobial oral biofilm development, environmental adaptation, and virulent factors regulation. Adhesins and receptors-mediated binding benefits microbial community residents by providing a broader habitat range and influences the temporal and spatial formation of highly organized polymicrobial community architecture. Furthermore, the close physical association allows residents to obtain metabolic support from their neighboring species. Cooperative metabolic interactions either via cross-feeding or through cooperatively metabolizing substrate maximize co-residents’ potential to extract energy from limited substrates. In addition to synergistic interactions, oral bacterial species are also engaged in intense competition for limited space and nutritional resources using compounds such as bacteriocin and H_2_O_2_, which plays a crucial role in defining the structure and activity of oral microbial communities. Intercellular signaling within the same or between different bacterial species can be achieved by contact dependent mechanisms or mediated by diffusible signal molecules. These signaling events play significant roles in coordinating gene expression involved in microbial physiology and pathobiology. Among the secreted signaling molecules, AIPs and AI-2 appear to be involved in both intra-species quorum sensing and a variety of interspecies interactions across oral species. More comprehensive investigations of microbial intercellular interactions will shed light on the complexity of multispecies oral microbial communities and may provide novel approaches for controlling microbial community-based pathogenesis.

## Conflict of Interest Statement

Dr. Shi is a part time chief science officer of C3 Jian Inc., which has licensed technologies from UC regents.
